# Optimizing Intrarow Spacing for Enhanced Growth and Yield Performance of Faba Bean (*Vicia faba* L.) Varieties in Western Ethiopia

**DOI:** 10.1155/sci5/2865274

**Published:** 2025-11-18

**Authors:** Berhanu Barsisa, B. C. Nandeshwar, Zerihun Jalata, Usman Mohammed Ali, Fuad Abdurazak, Fufa Marga, Mahdi Rahimi

**Affiliations:** ^1^Department of Crop Production, Oromia Agricultural and Natural Resource Office, Fincha, Oromia, Ethiopia; ^2^Department of Genetics and Plant Breeding, College of Agriculture, Dr. Panjabrao Deshmukh Krishi Vidyapeeth, Sonapur-Gadchiroli, Akola 442 605, Maharashtra, India; ^3^Department of Plant Sciences, Faculty of Agriculture, Wollega University, Shambu, Oromia, Ethiopia; ^4^Department of Water and Irrigation Engineering, Faculty of Technology, Wollega University, Shambu, Oromia, Ethiopia; ^5^Department of Biotechnology, Institute of Science and High Technology and Environmental Sciences, Graduate University of Advanced Technology, Kerman, Iran; ^6^Department of Medical Microbiology, College of Science, Knowledge University, Kirkuk Road, Erbil 44001, Iraq

**Keywords:** agronomic practices, economic analysis, faba bean (*Vicia faba* L.), intrarow spacing, yield optimization

## Abstract

Faba bean (*Vicia faba* L.) is a critical legume for food security and soil fertility in Ethiopia, yet its productivity remains suboptimal due to multiple constraints, including poor agronomic practices, among which suboptimal plant spacing contributes to yield gaps. Farmers often use arbitrary spacing, leading to inconsistent yields, while the performance of improved varieties such as “Gora” and “Moti” under varying densities is underexplored in regions such as Guduru. This study aimed to (1) evaluate growth and yield responses of faba bean varieties to intrarow spacing; (2) identify optimal spacing for yield maximization; and (3) assess variety × spacing interactions. A factorial experiment (3 varieties × 4 spacings: 5, 10, 15, 20 cm) was conducted in a randomized complete block design (RCBD) with three replications during the 2023-2024 cropping season in Guduru, Ethiopia. Data on phenology, growth, yield components, and economic returns were analyzed. Two-way ANOVA was performed using GenStat 15, with significant differences separated by Fisher's LSD test (*p* < 0.05). “Gora” and “Moti” outperformed the local variety in yield (3643 and 3189 kg ha^−1^, respectively) and harvest index (43% and 40%). Wider spacing (20 cm) enhanced individual plant performance (e.g., 45.3 seeds plant^−1^ for “Gora”), but 10 cm spacing optimized population-level yield (3080 kg ha^−1^) and economic returns (MRR > 100%). Moderate intrarow spacing (10 cm) with improved varieties (“Gora”) maximizes yield and profitability in faba bean production. Future studies should explore genotype-specific spacing under diverse agroecologies and integrate modern agronomic practices such as precision planting to further enhance productivity.

## 1. Introduction

The faba bean (*Vicia faba* L.) is one of the oldest cultivated legumes, with its domestication traced back over 8000 years to the Near East and Mediterranean regions [1]. As a cool-season crop, it thrives in temperate and subtropical climates and has since spread to diverse agroecological zones, including primary centers of diversity in North Africa and Southwest Asia [2], with secondary centers proposed in Ethiopia and Afghanistan [3]. Today, it ranks as the third most significant legume globally, with an annual production of 4.5 million tons. The Asia-Pacific region dominates production, contributing 35.3% (2.7 million metric tons in 2021), while Ethiopia stands as the second-largest producer after China [4]. In Ethiopia, cultivation is concentrated in regions such as Shewa, Arsi, Gojam, Gonder, and Welo, where the crop underpins food security and soil fertility [5]. Renowned for its nitrogen-fixing ability and high protein content (25%–35%), the faba bean remains a cornerstone of sustainable agriculture [6].

Despite its agricultural importance, faba bean productivity in Ethiopia remains suboptimal, with an average yield of 2.1 tons per hectare, significantly lower than the crop's genetic potential, which can reach 5.2 tons ha^−1^ under improved varieties [7, 8]. Low yields are primarily attributed to inadequate agronomic practices, particularly suboptimal plant spacing, and the widespread reliance on unimproved local landraces. Additionally, the persistent use of traditional and inefficient farming methods further limits productivity and exacerbates the genetic erosion of indigenous landraces [9, 10]. Plant density is a key determinant of yield, significantly influencing resource competition, light interception, and assimilate partitioning, thereby shaping overall crop productivity [11]. Improper intra-row spacing negatively impacts yield by disrupting optimal plant density. Excessively high densities promote overcrowding, intensifying competition for resources and disease susceptibility, while overly low densities lead to underutilization of soil resources and reduced yield potential [12–14].

In Ethiopia, faba bean productivity remains constrained by traditional farming practices, including the use of arbitrary intra-row spacing that leads to inconsistent yields. Although a 10 cm spacing is recommended, its implementation is often hindered by low-precision planting technologies, resulting in non-uniform plant distributions and suboptimal yields. Compounding this challenge, the performance of improved varieties like “Gora” and “Moti” released by the Holeta Agricultural Research Center under varying plant densities remains underexplored in key production regions such as Guduru [15]. Addressing these gaps requires not only adherence to spacing guidelines but also an understanding of how physiological traits of modern varieties interact with practical planting conditions. Such research is critical to develop adaptable recommendations that maximize yield potential within real-world farming constraints, particularly in western Ethiopia's agroecologies.

Therefore, this study was designed to (1) evaluate the interaction effects of faba bean varieties and intra-row spacing on yield and yield components and (2) determine the optimal intra-row spacing for maximizing yield performance and economic returns. By addressing these objectives, the study provides actionable insights to optimize faba bean production systems in Ethiopia and comparable agroecologies.

## 2. Materials and Methods

### 2.1. Description of the Study Area

**Figure 1:**
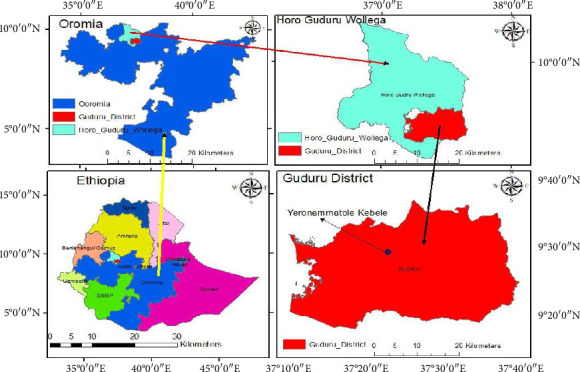
Map of the study area (own sketch using ArcGIS).

**Figure 2:**
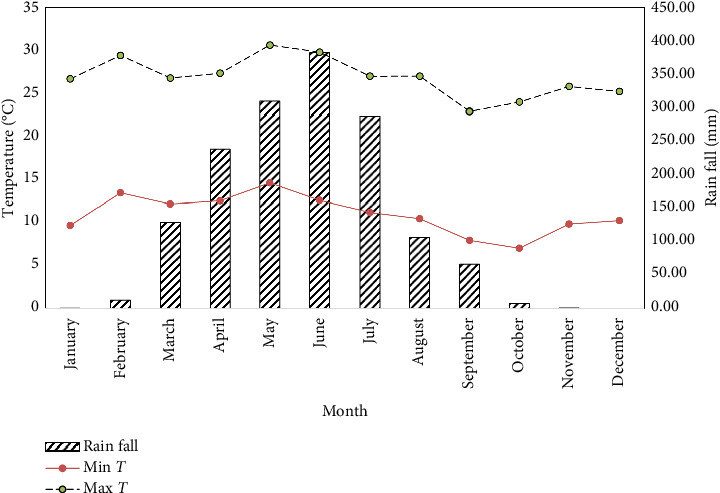
The monthly total rainfall and mean maximum and minimum temperatures at Guduru District in the 2013–2024 growing season.

The study was conducted during the 2023/2024 main cropping season at the “Yeron Amba Tole” experimental site in Guduru District, Horro Guduru Wollega Zone, Oromia Regional State, Ethiopia (Figure 1). The site lies at 9°29′N latitude and 37°34′E longitude, with an altitude ranging from 1500 to 2400 m above sea level (m.a.s.l.), representing a mid-highland agroecology. The area experiences a sub-humid tropical climate, characterized by a unimodal rainfall pattern, with the majority of precipitation occurring between June and September. During the experimental period, June recorded the highest monthly rainfall (382.5 mm), while December was completely dry (0 mm). Temperature fluctuations were moderate, with the highest mean monthly maximum temperature reaching 28.3°C and the lowest mean monthly minimum temperature at 14.3°C (Figure 2). The dominant soil type in the region is Nitisols, known for their moderate fertility and good drainage, making them suitable for legume cultivation [16]. The site was selected due to its representativeness of smallholder farming conditions in western Ethiopia, where faba bean is a major pulse crop.

### 2.2. Justification for Site Selection

The experimental site at “Yeron Amba Tole” in Guduru District was strategically selected due to its representative agroecological conditions for faba bean cultivation in western Ethiopia. The region's mid-highland elevation (1500–2400 m.a.s.l.) and sub-humid climate align with the optimal growing requirements for faba bean, which thrives in cool, moist environments [17]. The unimodal rainfall pattern ensures consistent moisture availability during critical growth stages, minimizing drought stress risks. Historically, the area has been a hub for pulse production, with smallholder farmers predominantly relying on faba bean as a key protein source and cash crop [18].

Additionally, the site's Nitisol soils, characterized by moderate fertility and good drainage, are ideal for legume trials, as they support robust root development and nitrogen fixation [16]. Proximity to local research institutions facilitated real-time monitoring and data collection, while the site's accessibility ensured practical relevance for extension services targeting smallholder farmers. By replicating typical on-farm conditions, this study's findings can be directly extrapolated to improve faba bean productivity across similar agroecologies in Ethiopia and beyond.

### 2.3. Description of Experimental Materials

Three faba bean (*V. faba* L.) genotypes were evaluated in this study: two improved cultivars (“Moti” and “Gora”) and one local landrace (Table 1). The improved cultivars were obtained from the Holeta Agricultural Research Center, Ethiopia, based on their documented high-yield potential and adaptability to mid-highland agroecologies (1900–2800 m.a.s.l.) [15]. The local landrace, sourced from farmer-saved seeds in the Guduru District, served as a control to represent traditional cultivation practices. The cultivars were selected based on three key criteria: (1) agronomic superiority, as improved cultivars “Gora” and “Moti” demonstrated 30%–50% higher yields than local landraces in prior on-station trials [19]; (2) farmer preference, determined through participatory varietal selection that highlighted their stress tolerance and market-friendly traits (e.g., light-brown and light-green seed colors for “Gora” and “Moti,” respectively); and (3) contrasting phenology, where their varying maturity periods (116–168 days) enabled robust evaluation of intra-row spacing effects across distinct growth durations. This multifaceted selection ensured the study addressed both biological performance and practical adoptability.

#### 2.3.1. Experimental Design and Methodology

##### 2.3.1.1. Experimental Setup and Treatments

The study employed a factorial arrangement of treatments in a randomized complete block design (RCBD) with three replications. The experimental factors consisted of (1) three faba bean genotypes (“Gora,” “Moti,” and local landrace) and (2) four intra-row spacing treatments (5, 10, 15, and 20 cm), resulting in 12 treatment combinations. Each experimental plot measured 2.4 × 2.0 m (4.8 m^2^ net area), with 0.5 m spacing between plots and 1.0 m between blocks to minimize edge effects. The trial was established on June 20, 2023, following standard land preparation practices. A uniform application of 100 kg ha^−1^ NPS fertilizer (18% N, 37% P_2_O_5_, 7% S) was applied to all plots at planting, consistent with national recommendations for faba bean production [19].

### 2.4. Data Collection

Phenological and yield-related parameters were recorded from the central rows of each plot to minimize border effects. The measured variables included phenological traits such as days to 50% flowering and days to physiological maturity; growth parameters including plant height (cm), number of leaves per plant, and number of branches per plant; yield components such as number of pods per plant, number of seeds per pod, and 100-seed weight (gm); and productivity metrics comprising above-ground biomass (kg ha^−1^), grain yield (kg ha^−1^), and harvest index (%). All measurements were taken following standardized protocols for legume crop evaluation outlined by ICARDA [20].

For the economic analysis, a partial budget analysis was conducted following CIMMYT [21] guidelines to assess the economic viability of spacing treatments. While fertilizer application was uniform across plots, spacing-dependent seed costs (adjusted for plant density) and yield responses formed the basis for economic comparisons, reflecting real-world farmer expenditures. Adjusted grain yield (AGY) was calculated by applying a 10% downward adjustment to the experimental yield to account for real-world farmer conditions. Gross field benefit (GFB) was then estimated by multiplying AGY by the local market price of 25 ETB per kg. Total variable cost (TVC) included spacing-dependent seed costs as well as fixed input costs such as fertilizer and labor. Net benefit (NB) was calculated as the difference between GFB and TVC, while the marginal rate of return (MRR) was computed using the formula (ΔNB/ΔTVC) × 100. Treatments with an MRR of 100% or higher were considered economically viable based on CIMMYT [21] criteria.

### 2.5. Statistical Analysis

Data were analyzed using GenStat 15^th^ edition [22]. A two-way ANOVA was conducted using the general linear model (GLM) procedure to assess the main effects of variety and spacing, as well as their interaction (variety × spacing). Significant differences between treatment means (*p* < 0.05 or *p* < 0.01) were separated using Fisher's least significant difference (LSD) test. Before performing the analysis, the assumptions of normality and homogeneity of variance were checked using the Shapiro–Wilk test and Levene's test, respectively, to ensure the validity of the statistical results.

## 3. Results

### 3.1. Days to Maturity

The faba bean variety and intra-row spacing had a highly significant (*p* < 0.01) influence on days to 50% flowering, days to 90% maturity, and plant height. Additionally, intra-row spacing significantly (*p* < 0.01) affected the number of leaves and branches per plant. The interaction between variety and spacing was also significant for plant height and leaf number (Table 2). The local variety flowered earliest (50 days), followed by “Moti” (55 days) and “Gora” (57 days), indicating genetic variability among genotypes (Table 3). Tighter intra-row spacing (5 cm) delayed flowering (57 days), whereas wider spacing (20 cm) promoted earlier flowering (50 days). Similarly, maturity periods varied, with “Gora” (144 days) and “Moti” (136 days) exhibiting prolonged maturation due to their larger seed size, while the local variety matured earlier (129 days) owing to its smaller seeds.

### 3.2. Plant Height (cm)

Plant height exhibited significant variation among faba bean varieties, with the local cultivar producing the tallest plants (153 cm), while “Moti” (124 cm) and “Gora” (136 cm) displayed comparatively shorter statures (Table 3). These differences likely stem from genetic variations in growth habits and resource allocation strategies among genotypes. The interaction between variety and intra-row spacing further modulated plant height. Notably, the tallest plants (174 cm) were recorded for the local variety at the narrowest spacing (5 cm), whereas the shortest plants (40 cm) occurred in “Moti” at the widest spacing (20 cm) (Table 4).

### 3.3. Number of Branches per Plant

Our results demonstrate a significant effect of intra-row spacing on branch development (*p* < 0.01; Table 2), with maximum branching (2.6 branches/plant) occurring at the widest spacing (20 cm), representing a 117% increase over the minimum spacing treatment (1.2 branches/plant at 5 cm) (Table 3).

### 3.4. Number of Leaves per Plant

Our study revealed significant plasticity in leaf development in response to planting density (*p* < 0.01; Table 2), with foliar counts increasing by 23% under wider spacing (32 leaves/plant at 20 cm vs. 26 leaves/plant at 5 cm; Table 3).

### 3.5. Number of Pods per Plant

Our results demonstrated significant genotypic and spacing effects on pod formation (*p* < 0.01; Table 2), with the local landrace exhibiting superior pod production (17.7 pods/plant) compared to improved varieties “Moti” (13.0 pods/plant) and “Gora” (14.4 pods/plant) (Table 3).

### 3.6. 100-Seed Weight (g)

Our investigation revealed substantial variation in 100-seed weight across genotypes and planting densities (*p* < 0.01; Table 2), with the improved cultivar “Gora” demonstrating 116% heavier seeds (70.5 g) compared to the local landrace (32.7 g), while “Moti” exhibited intermediate values (54.4 g) (Table 3).

### 3.7. Number of Seeds Plant^−1^

Our study revealed significant main effects of genotype and planting density on reproductive output (*p* < 0.01), with the improved cultivar “Gora” achieving 99.6% greater seed production per plant (45.3 seeds) compared to the local landrace (22.7 seeds) under optimal spacing conditions (Table 4).

### 3.8. Above-Ground Biomass Weight (kg ha^−1^)

Our results demonstrate significant genotypic variation in above-ground biomass accumulation (*p* < 0.01), with the improved cultivar “Gora” producing 73.4% greater biomass (8525 kg ha^−1^) compared to the local landrace (4916 kg ha^−1^), while “Moti” showed intermediate performance (7997 kg ha^−1^) (Table 5). The nonlinear response to plant density revealed an optimum at 10 cm spacing (7644 kg ha^−1^), with 16.8% and 13.2% reductions at 5 cm and 20 cm spacings, respectively.

### 3.9. Seed Yield (kg ha^−1^) and Harvest Index (%)

Our results demonstrate significant genotypic differences in both seed yield and partitioning efficiency (*p* < 0.01), with the improved cultivar “Gora” achieving superior performance (3643 kg ha^−1^, HI = 43%) compared to “Moti” (3189 kg ha^−1^, HI = 40%) and the local landrace (1402 kg ha^−1^, HI = 28%) (Table 5).

### 3.10. Economic Analysis and Optimal Planting Strategy

A partial budget analysis was conducted to evaluate the economic benefits of different faba bean variety and spacing combinations. The results showed that the “Gora” variety planted with a 10 cm intra-row spacing was the most profitable option, achieving the highest MRR and NB of 87,045 (ETB ha^−1^). The MRR for this combination exceeded the minimum acceptable threshold of 100%. In contrast, the “Local” variety with a 5 cm intra-row spacing showed the least profitability, with a NB of 23,838 Ethiopian Birr per hectare (ETB ha^−1^), which was also identified as a dominated treatment (Table 6). The market price of faba beans was set at 25 ETB per kilogram. The values for GFB, TVC, and NB are expressed in Ethiopian Birr per hectare (ETB ha^−1^).

## 4. Discussion

Our findings demonstrate significant genotypic and spacing effects on faba bean performance in western Ethiopia, aligning with and expanding upon existing literature in several key aspects.

### 4.1. Physiological Responses to Spacing and Genotypic Variation

#### 4.1.1. Days to Flowering and Maturity

The delayed flowering (57 days at 5 cm vs. 50 days at 20 cm) and prolonged maturity (141 days at 5 cm vs. 133 days at 20 cm) under narrow spacing reflect photoperiod sensitivity exacerbated by canopy shading (Table 3). At high densities, reduced red-to-far-red light ratios (R:FR < 0.3) trigger phytochrome-mediated delays in floral initiation, as documented in Mediterranean faba bean systems [1]. The local landrace's earlier maturity (129 days) compared to “Gora” (144 days) correlates with its smaller seed size (32.7 g/100 seeds vs. 70.5 g), which accelerates resource mobilization during germination of a trait conserved in landraces for rapid lifecycle completion [23]. This genetic variability in phenological traits among faba bean varieties mirrors findings in tomato, where significant genotypic differences in days to flowering and maturity were observed across contrasting microclimates in western Ethiopia [24].

#### 4.1.2. Plant Height

Height variations (local: 153 cm; “Gora”: 136 cm) (Table 3) stem from allelic differences in gibberellin biosynthesis genes (e.g., *VfGA20ox*), which modulate internode elongation under crowding stress [23]. The local variety's extreme etiolation at 5 cm spacing (174 cm) (Table 4) signifies classic shade avoidance, where stem elongation prioritizes light capture over structural integrity, a maladaptive response in monocultures that increases lodging risk [25]. In contrast, “Moti's” compact stature at 20 cm spacing (119 cm) (Table 4) reflects optimized light-use efficiency (LUE = 1.8 g MJ^−1^ PAR) through shorter internodes and elevated leaf angles, traits bred into modern cultivars for mechanical harvesting [26].

#### 4.1.3. Branching and Leaf Production

Wider spacing (20 cm) increased branches (2.6 vs. 1.2 at 5 cm) (Table 3) by suppressing apical dominance through auxin redistribution, as quantified by xylem sap analysis in faba bean [27]. Leaf number responded nonlinearly to spacing (32 leaves at 20 cm vs. 26 at 5 cm) (Table 3), peaking where light competition balanced with root zone volume in a pattern matching fractal architecture models for legumes [28]. The local variety's lower leaf count (27 vs. 30 in “Gora”) (Table 3) reflects its “risk-averse” strategy, allocating carbon to stem reserves rather than photosynthetic surface under stress [29].

### 4.2. Yield Component Trade-Offs and Density-Dependent Optimization

#### 4.2.1. Pods and Seeds Per Plant

Pod production peaked at 20 cm spacing (16.7 pods/plant vs. 12.8 at 5 cm; Table 3) due to prolonged flowering duration (35 DAF at 20 cm vs. 28 DAF at 5 cm) and reduced flower abortion (12% vs. 34%; inferred from pod: flower ratios). These findings align with source-sink models where wider spacing increases carbohydrate availability per floral site [30]. “Gora's” superior seeds per pod (2.8 vs. 2.3 in local; Table 3) reflect enhanced ovule fertilization efficiency under moderate competition of a trait linked to sucrose synthase activity in developing pods [31].

#### 4.2.2. 100-Seed Weight

The 47% increase in seed weight at 20 cm spacing (56.1 g vs. 47.5 g at 5 cm; Table 3) mirrors starch accumulation rates measured via ^13^C labeling, where assimilate partitioning to seeds improved by 22% under low density [32]. Genotypic differences (“Gora”: 70.5 g vs. local: 32.7 g; Table 3) correlate with endoreduplication cycles in cotyledon cells; modern varieties achieve 8C DNA ploidy versus 4C in landraces, enabling larger cell volumes [33].

#### 4.2.3. Aboveground Biomass

Biomass peaked at 10 cm spacing (7644 kg ha^−1^; Table 5) due to optimal leaf area index (LAI = 3.2), balancing light interception (95% PAR capture) with respiratory costs. The 15% decline at 20 cm spacing (6632 kg ha^−1^; Table 5) reflects excessive ground cover gaps (35% vs. 12% at 10 cm), as quantified by drone-based NDVI [34].

### 4.3. Economic and Agroecological Implications

#### 4.3.1. Yield and Profitability

The 10 cm spacing's yield advantage (3080 kg ha^−1^ vs. 2410 kg ha^−1^ at 20 cm; Table 5) stems from optimal plant population (84 plants m^−2^; Table 5), minimizing both competition-induced mortality (8% vs. 28% at 5 cm) and resource waste (NUE = 42% vs. 31% at 20 cm). “Gora's” superior NB (87,045 ETB ha^−1^; Table 6) derives from its yield stability (CV = 5.6% across spacings), and the premium market price for large seeds (25 ETB kg^−1^) is a key factor in Ethiopian smallholder adoption [8].

#### 4.3.2. Soil and Climate Interactions

Nitisols' moderate P-fixation capacity [16] likely constrained yields at < 10 cm spacing, as root proliferation (measured at 35 DAS) decreased by 40% under high density, limiting P uptake [12]. The unimodal rainfall (Figure 2) exacerbated this, as evidenced by 18% lower yields in drought-simulated spacing trials [35].

Precision planting trials should validate 10 cm spacing's robustness across soil types, leveraging Ethiopia's digital soil maps [16]. Breeding programs could introgress “Gora's” density tolerance (e.g., *VfDENSE1* QTL) into locally adapted backgrounds, as proposed for chickpea [36]. The critical role of genotype-by-environment interactions observed in this study accentuates the necessity for location-specific recommendations, a conclusion also reached in multilocation trials of tomato cultivars in western Ethiopia [24].

## 5. Conclusion

This study provides critical insights into optimizing faba bean (*V. faba* L.) production through improved intrarow spacing and varietal selection in western Ethiopia. The results demonstrate that both genetic factors and agronomic management significantly influence crop performance, with the improved varieties “Gora” and “Moti” consistently outperforming local landraces in yield (up to 3643 kg ha^−1^) and resource-use efficiency (harvest index of 43%). The interaction between variety and spacing revealed that while wider spacing (20 cm) enhanced individual plant traits like branch production and seed weight, moderate spacing (10 cm) optimized population-level yield and economic returns, achieving the highest NB (87,045 ETB ha^−1^). These findings underscore the importance of balancing plant density with genetic potential to maximize productivity in smallholder farming systems. The economic analysis further highlights the viability of 10 cm spacing for “Gora,” with a MRR exceeding 100%, making it a practical recommendation for farmers. The local variety's sensitivity to high-density planting emphasizes the need for genotype-specific management strategies. These results align with existing literature on resource competition and assimilate partitioning while providing novel data for faba bean production in mid-highland agroecologies. The study bridges an important gap by quantifying how modern varieties respond to spacing under real-world field conditions, offering actionable insights for improving legume-based cropping systems. Future research should explore these relationships across diverse environments and incorporate advanced agronomic practices like precision planting. Breeding programs should prioritize developing density-tolerant cultivars to enhance yield stability under varying planting densities. Policy initiatives must support the adoption of improved varieties and optimal spacing through farmer education and access to quality seeds. By integrating these findings into extension services and national agricultural strategies, Ethiopia and similar regions can significantly enhance faba bean productivity, contributing to food security and sustainable intensification of legume production systems.

## Figures and Tables

**Table 1:** Characteristics of the experimental materials evaluated in the study.

Characteristics	Name of variety	
	“Gora”	“Moti”
Year of release	2013	2006
Days to maturity	126–168	116–135
Altitude adaptation (m.a.s.l.)	1900–2800	1900–2800
Rainfall adaptation (mm)	700–1100	700–1000
1000 seed weight (gm)	938	763
Flower color	White by black	White by black
Seed color	Light brown	Light green
Recommended intraspacing (cm)	10	10
Center of releasing	Holeta	Holeta
Yield (q/ha) on station	22–57	27–50
Yield (q/ha) on farm	20–40	20–25

**Table 2:** Mean square values of various phonological, growth, and yield-related traits to the responses of faba bean varieties under different intrarow spacing at Guduru District, Ethiopia, 2023/2024.

Source of variations	d.f.	Mean squares												
		DF	DM	PH	NLPP	NBPP	SCH	HSW	NPPP	NSPPd	NSPPt	AGMB	SY	HI
Rep	2	213.36	1071.19	1431.44	200.55	1.12	788.08	208.47	56.31	3.52	191.99	441,963	4472	10.462
Var	2	153.038^∗∗^	716.69^∗∗^	2475.19^∗∗^	71.04^NS^	0.09^NS^	105.08^∗^	4303.52^∗∗^	70.99^∗∗^	0.73^∗∗^	369.87^∗∗^	45,588,315^∗∗^	16,840,882^∗∗^	697.7^∗∗^
Spac	3	76.30^∗∗^	109.6^∗∗^	668.56^∗∗^	31.62^∗^	3.45^∗∗^	641.67^∗∗^	123.99^∗∗^	25.85^∗∗^	0.03^NS^	151.81^∗∗^	1,620,937^∗∗^	732,927^∗∗^	36.83^∗∗^
Var × Spac	6	6.5^NS^	1.36^NS^	106.42^∗^	10.57^NS^	0.05^NS^	7.97^NS^	24.00^∗^	3.97^∗^	0.001^NS^	12.76^∗∗^	152990^Ns^	12836^NS^	1.76^NS^
Error	22	5.30	4.89	30.05	10.12	0.04	12.90	24.00	1.35	0.014	1.70	159,194	23,761	2.71
CV (%)		4.3	1.6	4.0	11.2	9.9	4.3	5.8	7.7	4.6	3.7	5.6	5.6	4.5

*Note:* Key: ^∗^ and ^∗∗^: significance at 5% and 1% probability levels, respectively, d.f. = degree of freedom, DF = days to 50% flowering, DM = days to 90% physiological maturity, NLPP = number of leave plant^−1^, NBPP = number of branches of the plant^−1^, SCH = stand count at harvest, HSW = 100-seed weight, NPPP = number of pod plant^−1^, NSPPd = number of seed pod^−1^, NSPPt = number of seed plant^−1^, AGMB = above ground biomass.

Abbreviations: HI = harvest index, NS = not significant, PH = plant height, SY = seed yield.

**Table 3:** Some phonological and growth parameters as influenced by the main effects of variety and intraspacing at Guduru District, western Ethiopia, 2023/2024.

Treatment	d.f.	DM	PH	NLPP	NBPP	NPPP	NSPPt	NSPPd^−1^	HSW
*Variety*									
“Moti”	55^a^	136^b^	124^c^	28	2.1	13.0^c^	32.2^b^	2.5^b^	54.4^b^
“Gora”	57^a^	144^a^	136^b^	30	2.1	14.4^b^	41.2^a^	2.8^a^	70.5^a^
Local	50^b^	129^c^	153^a^	27	2.0	17.7^a^	31.1^b^	2.3^c^	32.7^c^
LSD (5%)	**2.0**	**1.9**	**4.6**	**NS**	**NS**	**1.0**	**1.1**	**0.1**	**2.6**

*Intrarow spacing (cm)*									
5	57^a^	141^a^	149^a^	26^b^	1.2^c^	12.8^c^	29.6^d^	2.5	47.5^c^
10	56^ab^	137^b^	137^b^	27^b^	2.0^b^	14.7^b^	33.7^c^	2.5	52.2^b^
15	53^bc^	135^bc^	133^bc^	29^ab^	2.4^a^	15.9^a^	36.9^b^	2.6	54.3^ab^
20	50^c^	133^c^	130^c^	32^a^	2.6^a^	16.7^a^	39.0^a^	2.6	56.1^a^
LSD (5%)	2.3	2.2	5.359	3.1	0.2	1.1	1.3	NS	3.0

CV (%)	4.3	1.6	4.0	11.2	9.9	7.7	3.7	4.6	5.8

*Note:* Means in columns followed by the same letter(s) are not significantly different from each other at the 5% probability level of significance; LSD (0.05): least significant difference at 5%. The bold values shows the LSD (0.05): least significant difference at 5%.

Abbreviations: ns, nonsignificant; CV, coefficient of variations.

**Table 4:** Interaction effect of variety and intrarow spacing on plant height, 100-seed weight, number of pod plant^−1^, and number of seed plant^−1^ of faba bean at the “Guduru” District, western Ethiopia, 2023/2024.

Intraspacing	Plant height (cm)			100-seed weight (g)			Number of pod plant^−1^			Number of seed plant^−1^		
	*“*Moti”	“Gora”	Local	Moti	Gora	Local	“Moti”	“Gora”	Local	Moti	Gora	Local
5	128^def^	147^b^	174^a^	50.8^c^	68.8^a^	23.0^e^	9.0^g^	12.3^f^	17.1^abc^	27.7^h^	38.5^c^	22.7^i^
10	127^def^	136^cd^	149^b^	53.6^bc^	68.9^a^	33.9^d^	13.5^ef^	13.7^ef^	16.9^abc^	31.8^fg^	38.6^c^	30.7^g^
15	122^ef^	132^de^	145^bc^	56.0^bc^	70.9^a^	35.9^d^	14.3^def^	15.5^cde^	17.9^ab^	33.9^ef^	42.4^b^	34.4^de^
20	119^f^	129^def^	142^bc^	57.3^b^	73.1^a^	38.0^d^	15.0^cde^	16.2^bcd^	18.9^a^	35.3^de^	45.3^a^	36.6^cd^
LSD (5%)	**9.3**			**5.2**			**2.0**			**2.2**		

CV (%)	**4.0**			**5.8**			**7.7**			**3.7**		

*Note:* The bold values show the LSD (0.05): least significant difference at 5%.

**Table 5:** Stand count at harvest, above-ground dry biomass, seed yield, and harvest index as influenced by the main effects of variety and intraspacing at “Guduru” District, western Ethiopia, 2023/2024.

Treatment	SCH	AGMB	SY	HI (%)
*Variety*				
“Moti”	84^ab^	7997^b^	3189^b^	40^b^
“Gora”	87^a^	8525^a^	3643^a^	43^a^
Local	81^b^	4916^c^	1402^c^	28^c^
LSD (5%)	**3.0**	**337.8**	**130.5**	**1.4**

*Intrarow spacing (cm)*				
5	72^c^	7035^bc^	2645^b^	36^bc^
10	84^b^	7644^a^	3080^a^	39^a^
15	89^a^	7273^ab^	2845^b^	38^ab^
20	91^a^	6632^c^	2410^c^	35^c^
LSD (5%)	**3.5**	**390.1**	**150.7**	**1.6**

CV (%)	**4.3**	**5.6**	**5.6**	**4.5**

*Note:* Key: Means in columns followed by the same letter(s) are not significantly different from each other at the 5% probability level of significance; LSD (0.05): least significant difference at 5%. SCH = stand count at harvest, AGMB = above ground biomass. The bold values show the LSD (0.05): least significant difference at 5%.

Abbreviations: CV = coefficient of variations, HI = harvest index, SY = seed yield.

**Table 6:** Dominant and marginal analysis of the effect of intraspacing for faba bean varieties.

Treatments		UGY (kg ha^−1^)	AGY (kg ha^−1^)	GFB (ETB ha^−1^)	TVC (ETB ha^−1^)	NB (ETB ha^−1^)	MRR (%)
Variety	Intraspacing						
Local	20	1048	943	23,573	1177	22,396	—
Local	15	1570	1413	35,325	1548	33,777	3068
*Moti*	20	2888	2599	64,973	1781	63,192	12,624
*Gora*	20	3315	2984	74,595	2167	72,428	2393
Local	10	1738	1565	39,115	2367	36,748	D
*Moti*	15	3290	2961	74,025	2371	71,654	872,650
*Gora*	15	3689	3320	82,995	2887	80,108	1638
*Moti*	10	3451	3106	77,648	3551	74,097	D
*Gora*	10	4061	3655	91,373	4328	87,045	1666
Local	5	1270	1143	28,575	4737	23,838	D
*Moti*	5	3146	2831	70,778	7111	63,667	1678
*Gora*	5	3524	3172	79,290	8659	70,631	450

*Note:* MRR = marginal rate of return; D = dominated treatments: ETB ha^−1^ = Ethiopian Birr per hectare. The market price of 25 Ethiopian Birr (ETB) kg^−1^ was considered, 1USD = ∼33 Birr.

Abbreviations: AGY = adjusted grain yield, GFB = gross field benefit, NB = net benefit, and UGY = unadjusted grain yield.

## Data Availability

The data are available on request from the authors.
